# Romosozumab was not effective in preventing multiple spontaneous clinical vertebral fractures after denosumab discontinuation: A case report

**DOI:** 10.1016/j.bonr.2020.100288

**Published:** 2020-06-05

**Authors:** Masafumi Kashii, Kosuke Ebina, Kazuma Kitaguchi, Hideki Yoshikawa

**Affiliations:** a Department of Orthopedic Surgery, Toyonaka Municipal Hospital, Toyonaka, Osaka, Japan; b Department of Musculoskeletal Regenerative Medicine, Graduate School of Medicine, Osaka University, Suita, Osaka, Japan

**Keywords:** Denosumab, Romosozumab, Vertebral fractures, Discontinuation

## Abstract

Discontinuation of denosumab is associated with the increase of bone turnover markers to above-baseline levels (so-called rebound in bone turnover) and rapid bone loss. Several studies have reported vertebral fractures (VFs), particularly multiple spontaneous clinical VFs (MSCVFs), occurring after discontinuation of denosumab. There is currently no recommendation for the management of VFs including MSCVFs. Presently, romosozumab is the strongest anti-osteoporotic agent that inhibits sclerostin and rapidly increases bone mass, but it is uncertain that romosozumab is an effective treatment choice to treat VFs occurring after discontinuation of denosumab. Herein we reported a novel case of a 60-year-old woman who was treated with romosozumab after discontinuation of denosumab and subsequently suffered MSCVFs under romosozumab treatment. She had a history of two osteoporotic VFs (T6 and T8) and received five doses of 60 mg denosumab every 6 months following the osteoporosis diagnosis. As per the patient's convenience, the sixth denosumab injection was postponed. To improve the persistent low bone mass in the lumbar spine (T-score −3.8), 210 mg romosozumab was administered monthly after 9 months following the last denosumab injection. At the first romosozumab injection, she had no clinical symptoms such as low back pain, but her bone formation and resorption marker levels elevated compared with those treated with denosumab. After three doses of romosozumab, spontaneous severe low back pain occurred, and time-course radiographs revealed five new VFs (T12, L2, L3, L4, and L5). Romosozumab administration had no suppressive effect on bone resorption during the rebound in bone turnover after discontinuation of denosumab. This case suggests that romosozumab is not effective in preventing VFs or MSCVFs after denosumab discontinuation.

## 1 Introduction

Bisphosphonates remain in the skeletal bone even after their discontinuation because of their high affinity to skeletal bone (Meier et al., 2017). However, the clinical benefit of other anti-osteoporosis agents, except bisphosphonates, diminishes over time following discontinuation. Discontinuation of denosumab has been known to be associated with the increase of bone turnover markers to above-baseline levels (rebound in bone turnover) and rapid bone loss (Bone et al., 2011; Popp et al., 2018), and recent reports have demonstrated vertebral fractures (VFs), particularly multiple spontaneous clinical VFs (MSCVFs), occurring after discontinuation of denosumab (Anastasilakis et al., 2017; Tripto-Shkolnik et al., 2018; Lamy et al., 2019). There is currently no recommendation for the management of VFs including MSCVFs (Lamy et al., 2019). A bone anabolic agent such as teriparatide seems to be an effective treatment choice to treat VFs occurring after discontinuation of denosumab. However, switching therapy from denosumab to teriparatide results in transient bone loss in the spine and hip with the rebound in the bone turnover (Leder et al., 2015).

Sclerostin is an antagonist for canonical Wnt/β-catenin signaling that regulates bone mass, and it is a therapeutic target for the treatment of osteoporosis (Delgado-Calle et al., 2017). Romosozumab, which became available in March 2019, is a monoclonal anti-sclerostin antibody that rapidly increases bone mass through the following dual effects on the bone: increasing bone formation and decreasing bone resorption (Padhi et al., 2011; McClung et al., 2014). In a randomized controlled trial that compared romosozumab with placebo, treatment with romosozumab for 12 months showed a dramatic increase in bone mass and significant reduction of the risk of new VFs and clinical fractures among postmenopausal women with osteoporosis (Cosman et al., 2016). Furthermore, treatment with romosozumab is reported to significantly increase bone mass among women with postmenopausal osteoporosis transitioning from alendronate treatment (Langdahl et al., 2017). The second course of romosozumab transitioning from treatment with denosumab increased bone mass in the spine, maintained bone mass in the hip, and was well tolerated (Kendler et al., 2019). Presently, romosozumab is the strongest anti-osteoporotic agent that rapidly increases bone mass when compared with other agents, but it is uncertain that romosozumab is an effective treatment choice to treat VFs or MSCVFs occurring after discontinuation of denosumab.

To the best of our knowledge, this is the first reported case of a 60-year-old woman who was treated with romosozumab after discontinuation of denosumab and subsequently suffered MSCVFs under romosozumab treatment.

## 2 Case presentation

**Fig. 1:**
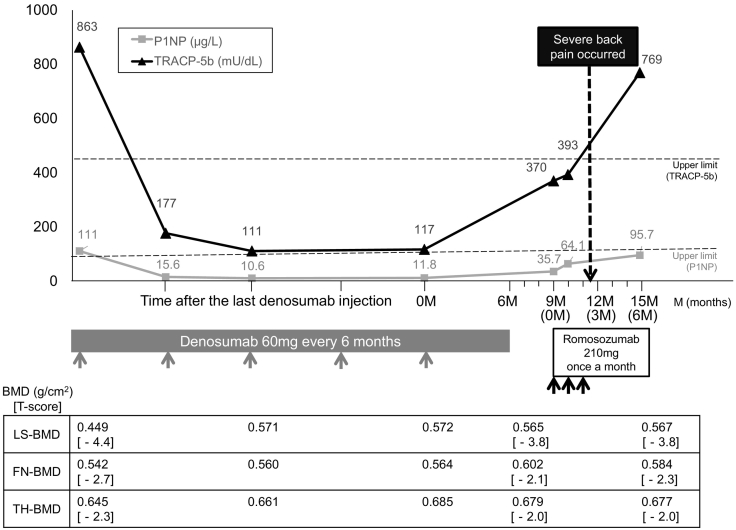
Treatment progress of switching from denosumab to romosozumab with changes of bone turnover markers and bone mineral density (BMD) in the lumbar spine and hip. *FN* femoral neck, *LS* lumbar spine, *P1NP* intact N-terminal propeptide of type I procollagen, *TH* total hip, *TRACP-5b* tartrate-resistant acid phosphatase 5b.

**Fig. 2:**
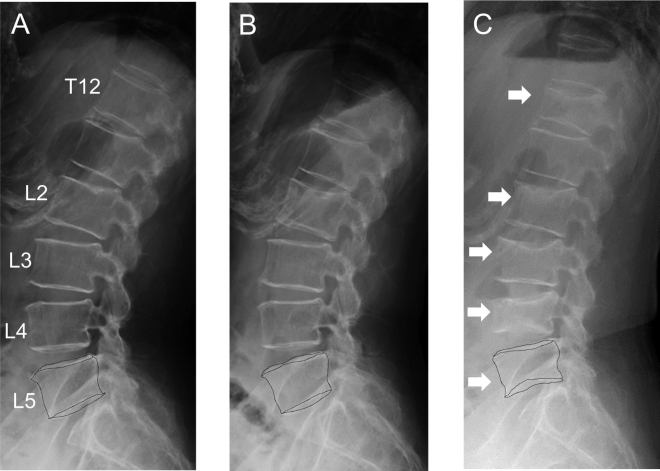
Lateral spine radiographs with the patient standing. (A) Before romosozumab treatment, (B) 2 months after romosozumab treatment, (C) 4 months after romosozumab treatment. Time-course radiographs revealed five new vertebral fractures with vertebral deformity (T12, L2, L3, L4, and L5). Dot lines show the outline of L5 vertebral body.

This 60-year-old woman was firstly diagnosed with osteoporosis at the age of 57. She had a history of two VFs in the thoracic spine (T6 and T8) after lifting a heavy load. She had no other comorbidities such as cardiovascular diseases, osteomalacia, hyperthyroidism, and hyperparathyroidism. She also took no medicines which induce medication-related osteoporosis. During her first hospital visit, dual-energy absorptiometry (DXA) revealed severe osteoporosis with low bone mineral density (BMD) in the lumbar spine (T-score of −4.4) and low BMD in the femoral neck (T-score of −2.7) (Fig. 1). Initially, we strongly recommended the usage of teriparatide to increase the lumbar spine BMD, but she felt uncomfortable with self-injection. As a result, 60 mg denosumab was subcutaneously administered every 6 months, which prevented further fractures. After five denosumab injections, her lumbar spine BMD increased by 26%; however, no increase was observed within the past year (Fig. 1). Before the sixth denosumab injection in April 2019, she became sick and could not visit our hospital. Unfortunately, denosumab treatment was discontinued although we instructed her not to miss her routine visit every 6 months. The switching therapy from denosumab to romosozumab is not a standard treatment, but romosozumab was recommended in order to improve the persistent low bone mass in lumbar spine BMD. There was a 9 month-interval between the last denosumab administration and the first romosozumab administration; 210 mg Romosozumab was administered monthly since July 2019 (Fig. 1). At the first romosozumab injection, she had no clinical symptoms such as low back pain, but she experienced spontaneous severe low back pain after three doses of romosozumab. Spine radiographs did not show any vertebral deformities just after she complained severe back pain (Fig. 2B). Due to claustrophobia, earlier diagnosis using magnetic resonance images could not be performed; however, time-course radiographs revealed five new VFs (at the T12, L2, L3, L4, and L5) (Fig. 2C). Spine radiographs revealed MSCVFs and denosumab treatment was resumed after discontinuation of romosozumab.

At the first presentation, blood and urine tests were performed to rule out secondary osteoporosis and other bone diseases. Her serum alkaline phosphatase level was high (612 IU/L) due to bone healing after two thoracic VFs. The levels of serum calcium, blood urea nitrogen, creatinine, estimated glomerular filtration rate, and intact parathyroid hormone were within the normal range. Her serum intact N-terminal propeptide of type I procollagen (P1NP) (standard range, 26.4–98.2 μg/L) was high (111 μg/L) due to bone healing after two thoracic VFs. The tartrate-resistant acid phosphatase 5b (TRACP-5b) (standard range, 120–420 mU/dL) level was also high (863 mU/dL) (Fig. 1). During the treatment with denosumab, serum P1NP and TRACP-5b levels were lower, suggesting that denosumab efficiently suppresses bone turnover. However, discontinuation of denosumab for 3 months induced the rebound in bone turnover with the rapid increase of serum P1NP and TRACP-5b levels (P1NP, 35.7 μg/L and TRACP-5b, 370 mU/dL). At the second romosozumab injection, the serum TRACP-5b level did not decrease (before the first injection, 370 mU/dL; before the second injection, 393 mU/dL). After six doses of romosozumab, DXA revealed no increased lumbar spine BMD when compared with that obtained during the first initiation of romosozumab administration (Fig. 1). Serum P1NP and TRACP-5b levels were noted to be even higher (P1NP, 95.7 μg/L and TRACP-5b, 769 mU/dL) at 6 months after the treatment with romosozumab (Fig. 1).

## 3 Discussion

Discontinuation of denosumab is associated with the rebound in bone turnover and rapid bone loss (Bone et al., 2011; Popp et al., 2018). The incidence of VFs after denosumab discontinuation is uncertain; however, MSCVFs frequently occur (≥1/100 and <1/10) in patients not treated with bisphosphonate (Uebelhart et al., 2017). In a recent review, 70 patients (69 women and one man; average age, 67.3 years) experienced 399 spontaneous VFs (median 5) within 7 and 20 (median 11) months after their last denosumab injection (Lamy et al., 2019). Younger women tend to be at a higher risk of MSCVFs (Lamy et al., 2019). Several studies have argued that bisphosphonate treatment before the denosumab administration can possibly reduce the risk of MSCVFs, and a sequential treatment with bisphosphonates after discontinuation of denosumab can also possibly reduce the risk (Uebelhart et al., 2017; Tripto-Shkolnik et al., 2018; Lamy et al., 2019). The present patient, who experienced five spontaneous VFs 11 months after the last denosumab injection was relatively young and had not been previously treated with bisphosphonate. These characteristics highly agree with those reported by other studies (Anastasilakis et al., 2017; Tripto-Shkolnik et al., 2018; Lamy et al., 2019). We firmly believe that 9 months interval between the last denosumab injection and the initiation of romosozumab treatment induced the rebound in bone turnover and this patient experienced MSCVFs after discontinuation of denosumab, even under romosozumab treatment.

Presently, the management for VFs occurring after discontinuation of denosumab remains to be elucidated (Lamy et al., 2019). The management should be started in the early stages and earlier diagnosis is necessary. Magnetic resonance imaging (MRI) has a high degree of accuracy for making a definite diagnosis of acute VF. MRI is useful tool to detect VF in the early stages (Anastasilakis et al., 2020b). There is a possibility that repetitive MSCVFs in a patient who had previous MSCVFs after stopping denosumab (Niimi et al., 2020; Anastasilakis et al., 2020a). Delayed additional denosumab administration do not completely eliminate the risk of VFs or MSCVFs occurring after discontinuation of denosumab (Niimi et al., 2020). Bone anabolic agents such as teriparatide and romosozumab appear to be one of the treatment choices. However, both teriparatide and romosozumab as a sequential therapy after the treatment with denosumab temporarily decrease bone loss in the spine and hip (Leder et al., 2015; Kendler et al., 2019). A subsequent negative chain of multiple VFs was not prevented by romosozumab treatment in this patient. The activation of canonical Wnt/β-catenin signaling may decrease osteoclastogenesis both through osteoprotegerin produced by osteoblasts and through a direct effect on osteoclasts (Lerner and Ohlsson, 2015; Delgado-Calle et al., 2017). However, the serum TRACP-5b level in this case did not decrease at the second romosozumab injection, the time before she complained low back pain. This revealed that activation of canonical Wnt/β-catenin signaling by romosozumab cannot show its suppressive effect on bone resorption during the rebound in bone turnover after discontinuation of denosumab. Serum P1NP and TRACP-5b levels were even higher at 6 months after the treatment with romosozumab. It is thought that bone healing process after MSCVFs have greatly affected the increase of serum P1NP and TRACP-5b levels.

To the best of our knowledge, this is the first case report of a patient who experienced MSCVFs under romosozumab treatment after discontinuation of denosumab immediately after the availability of romosozumab in the market. However, no cases of MSCVFs occurring after switching from denosumab to teriparatide were reported. There are two possible reasons for this difference. 1) Physicians rarely use teriparatide after denosumab except in the case of osteonecrosis of jaw or atypical femoral fracture occurred under denosumab treatment. Teriparatide was put on the market at 2002 in USA earlier than denosumab, and the DATA-switch study showed the transient bone loss of switching therapy from denosumab to teriparatide (Leder et al., 2015). However, up to date there were no reported cases with MSCVFs after switching from denosumab to teriparatide. 2) The difference in the pharmacological action between teriparatide and romosozumab may be associated with MSCVFs. Lamy et al. argued that microdamage accumulation in the cancellous bone brought by extremely high bone turnover induce fractures in spine, which are rich in cancellous bone (Lamy et al., 2019). Teriparatide has been shown to accelerate fracture healing in animal studies (Mognetti et al., 2011; Shi et al., 2016), which has also been confirmed in humans (Kitaguchi et al., 2019). Anti-sclerostin antibody has also been reported to accelerate fracture healing in animal models (Ominsky et al., 2011); however, its effect on cancellous bone regeneration is weaker than that of teriparatide, which has more powerful effects on the mesenchymal stem cell differentiation in osteoblasts and adipocytokine inhibition (Agholme et al., 2014). The difference in repair ability for microcracks may be associated with the occurrence of MSCVFs.

## 4 Conclusion

This is the only a case report and it's difficult to draw a definitive conclusion from this case. However, the findings of this case suggested that romosozumab cannot demonstrate its strong dual effects on the bone during the rebound in bone turnover after discontinuation of denosumab. The least clinicians can do is to initiate the therapy at 6 months after the last denosumab injection when considering the switching therapy from denosumab to romosozumab.

## Transparency document

Transparency document Image 1

## Funding source

None.

## Declaration of competing interest

All authors declare that they have no conflicts of interest.
